# Smoking in Ghana: a review of tobacco industry activity

**DOI:** 10.1136/tc.2009.030601

**Published:** 2009-04-08

**Authors:** E Owusu-Dabo, S Lewis, A McNeill, S Anderson, A Gilmore, J Britton

**Affiliations:** 1 UK Centre for Tobacco Control Studies, Division of Epidemiology and Public Health, Division of Respiratory Medicine, University of Nottingham, Clinical Sciences Building, City Hospital, Nottingham, UK; 2 School for Health, University of Bath, Bath & London School of Hygiene and Tropical Medicine, University of London, London, UK; 3 Department of Community Health, School of Medical Sciences, College of Health Sciences, Kwame Nkrumah University of Science and Technology, Kumasi, Ghana

## Abstract

**Background:**

African countries are a major potential market for the tobacco industry, and the smoking epidemic is at various stages of evolution across the continent. Ghana is an African country with a low prevalence of smoking despite an active tobacco industry presence for over 50 years. This study explores potential reasons for this apparent lack of industry success.

**Objective:**

To explore the history of tobacco industry activity in Ghana and to identify potential reasons for the current low prevalence of smoking.

**Methods:**

A search was made of tobacco industry archives and other local sources to obtain data relevant to marketing and consumption of tobacco in Ghana.

**Findings:**

British American Tobacco, and latterly the International Tobacco Company and its successor the Meridian Tobacco Company, have been manufacturing cigarettes in Ghana since 1954. After an initial sales boom in the two decades after independence in 1957, the sustained further increases in consumption typical of the tobacco epidemic in most countries did not occur. Possible key reasons include the taking of tobacco companies into state ownership and a lack of foreign exchange to fund tobacco leaf importation in the 1970s, both of which may have inhibited growth at a key stage of development, and the introduction of an advertising ban in 1982. BAT ceased manufacturing cigarettes in Ghana in 2006.

**Conclusion:**

The tobacco industry has been active in Ghana for over 50 years but with variable success. The combination of an early advertising ban and periods of unfavourable economic conditions, which may have restricted industry growth, are likely to have contributed to the sustained low levels of tobacco consumption in Ghana to date.

Tobacco smoking caused an estimated 4.8 million deaths worldwide in 2000, half of them in developing countries.^1^ ^2^ This figure is likely to rise^3^ as a consequence of population growth, increased wealth,^4^ ^5^ erosion of social taboos preventing smoking among women and intensive marketing by multinational tobacco companies.^6^ ^7^ Consequently, developing countries will bear the brunt of the death and disability caused by tobacco smoking in the 21st century.^8^ ^9^ Many have committed to implement the World Health Organization’s (WHO) Framework Convention on Tobacco Control (FCTC),^10^ but this may prove difficult to achieve in practice. It is therefore crucial to study the evolution of the smoking epidemic in these countries to understand better the context for effective tobacco control policy implementation.

Ghana is a West African country with a population of 22 million (approximately 12th highest in Africa), an annual population growth of 2.17%, a gross national income (in 2005) of $450 per capita and current economic growth close to 6%.^7^ ^11^ ^12^ Formerly the Gold Coast, Ghana became independent in 1957 and after periods of military rule has been a stable democracy since 1992. Over the past two decades, Ghana has experienced strong economic growth in relation to most African countries.^13^ Ghana has thus been a prime target for tobacco marketing, particularly by British American Tobacco (BAT), which has been manufacturing cigarettes in Ghana for most of the past 50 years. However, while detailed smoking prevalence data are not available for Ghana, a representative national household survey recently estimated prevalence to be around 9% in men and under 1% in women,^14^ suggesting that the expected epidemic rise in smoking prevalence has not occurred. We have used industry archives to study the evolution of the tobacco industry in Ghana and to try to identify potential explanations for this low smoking prevalence.

## METHODS

We studied international tobacco industry documents released as a result of litigation in the US. We took an iterative approach, searching at first for terms such as Ghana, West Africa and Ghanaian, and then using additional search terms identified through these initial searches. The additional terms included BAT’s cigarette brand names used in Ghana and neighbouring countries, BAT UK & Export (BATUKE) and Britanique Ivoirienne Tabac Conseil (BRITCo). Names of BAT workers in Ghana and the West African Region and specific project names were also used, sometimes in combination, to understand the contexts within which they have been used. Searches were performed on the legacy Tobacco Documents library (http://legacy.library.ucsf.edu) and the British American Tobacco Documents Archive (http://bat.library.ucsf.edu) between March and October 2007. The majority of documents identified were BAT documents. A total of over 1000 documents were extracted into a database and sorted according to relevance and date. Following further analysis, approximately 300 documents were analysed in detail for the purpose of this work. We also obtained official cigarette production, import and export data (from the United Nations (UN) Food and Agriculture Organization, United States Department of Agriculture Foreign Agricultural Service and other relevant sources (http://www.usda.gov), (http://www.fao.org) to gain further insight into the likely scale of the tobacco market in Ghana and to estimate the extent of any unofficial trade.

Approval for the study was granted by the Committee of Human Research and Ethics of the School of Medical Sciences of the Kwame Nkrumah University of Science and Technology, Kumasi, Ghana and the Local Ethics Committee of the University of Nottingham, UK.

## RESULTS

### The tobacco industry in Ghana

Although tobacco has been used in the Gold Coast region for centuries,^15^ ^16^ major commercialisation did not begin until after World War II, when demand for cigarettes was increased by the return of Ghanaian servicemen who had taken up smoking while on war duty overseas. In 1948 BAT established cigarette distribution depots in partnership with local businesses,^15^ ^17^ ^18^ and in 1951 formed the Gold Coast Tobacco Company (GCTC) to manage this network. In 1952 BAT established the Pioneer Tobacco Company (PTC) to develop tobacco cultivation and cigarette manufacture.^19^ ^20^ Cigarette production began in 1954.^18^ ^21^ In 1959, 2 years after independence, PTC took over GCTC.^22^

PTC enjoyed strong support from the new Ghanaian Government at the time of independence; in 1957, President Kwame Nkrumah said:

“I should like to draw attention to an excellent example of the kind of investment we appreciate in this country. I refer to Pioneer Tobacco Company. Here is a young company working most efficiently among Africans, encouraging the growth of its raw materials…this is an excellent case scenario for private enterprise”.^19^

However, in 1962 the Government legislated to bring tobacco marketing under state control and bought the tobacco leaf department of PTC to create the Ghana Tobacco Leaf Company, with PTC retaining shares and management roles in the new company.^23^ ^24^ After Nkrumah was deposed in 1967, private enterprises flourished again until the Government mandated Ghanaian ownership of major foreign companies and organisations in 1976. The Government then took a 40% share of PTC, 15% was sold to the public and 45% retained by BAT.^23^ ^25^ BAT continued to provide management and other services to the company.^26^

PTC enjoyed monopoly status in Ghana until 1976, when International Tobacco Ghana (ITG) was established as a commercial partnership with Rothmans UK to produce Rothmans King Size cigarettes, which by that time had achieved a significant market share in Ghana through smuggling from Togo.^27^^–^31 However in 1989 the Ghana Customs Excise and Preventative Service (CEPS) served a levy on ITG for US $3.3 million in unpaid duty and sales tax, causing ITG to cease trading. Some months later, ITG’s assets were sold to the Meridian Tobacco Company (MTC), which was jointly owned by the state-owned Social Security and National Insurance Trust and Rothmans UK.^32^ ^33^

When BAT bought Rothmans in 1999, PTC merged with MTC to become British American Tobacco Ghana, thus recreating a manufacturing monopoly for BAT in Ghana. In 2006 the company recorded a total income of 260 565 million Ghanaian cedis (GHC; 1 GHC is approximately equal to 1 US dollar), an increase of 9.57% over the preceding year, but made a loss of over 11 million GHC (compared with a net profit of GHC 30 350 million in 2005).^34^ In December 2006 the company closed and manufacturing was relocated to Nigeria.^34^ ^35^

### Tobacco and cigarette production and consumption

**Figure 1:**
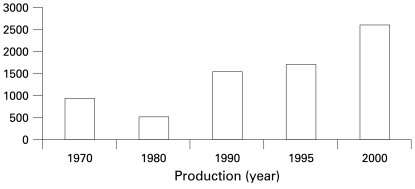
Tobacco leaf production in metric tons, Ghana. Source: European Research Council.^43^

By the end of 1966, according to BAT, cigarette sales in Ghana amounted to 200 million per month, and between 1968 and 1976 production increased by an estimated 76%.^36^ ^37^ However, growth in production was limited during this period by a lack of tobacco leaf; domestic production was insufficient, and importation was substantially limited by foreign exchange shortages.^36^ ^38^ ^39^ From 1980 however, domestic leaf production increased (fig 1),^40^ to the point that Ghana was able to start to export tobacco leaf in 1986.^18^ ^41^ ^42^

**Figure 2:**
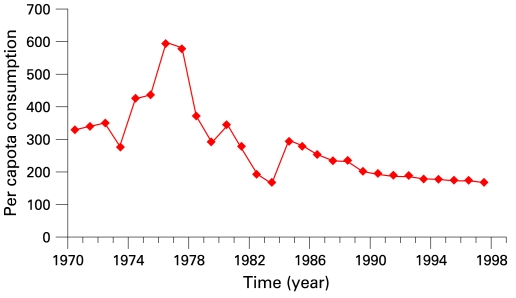
Annual per capita consumption of cigarettes, Ghana (1970–1997). Source: United Nations Commodity Trade^40^ (COMTRADE).

According to the UN African Regional Report, per capita consumption of cigarettes in Ghana also increased in the early 1970s to a peak of 600 per person in 1977,^40^ but this has since declined to a low of 171 per person in 1997^40^ (fig 2). Consumption estimates based on official production figures since the late 1990s are incomplete and are thought not to include consumption of smuggled cigarettes (see below), but demonstrate a slow but progressive decline in consumption since 1984,^43^ ^44^ although consumption appears to have increased immediately following the advertisement ban in 1982 (see below).

### Cigarette brands in Ghana

The first Ghanian-made cigarette, Tusker, was produced from imported tobacco in 1954; the second brand, Town Hall, was introduced in 1955.^45^^–^47 By 1979, there were 10 cigarette brands on the Ghanaian market, of which State Express 555 and Embassy (BAT) accounted for 70%, and Rothman’s king size and Pall Mall (ITG) 25%.^37^ ^45^ ^48^ By the time of closure of the BAT factory in 2006 there were over 25 cigarette brands on the Ghanaian market, most of them manufactured in Ghana by BAT but including Bond Street, Gold Seal and others brought from Togo and Nigeria.

### Price and taxation

In 1978 the 10 major cigarette brands in Ghana were produced with official or nominal retail prices in local currency equivalent to between US$0.67 and US$1.12.^37^ ^45^ However the real price at which packs retailed was considerably higher, at between US$1.49 and US$3.72.^37^ By 1987 however, BAT market survey data indicate that popular brands were selling for the equivalent of $0.83 to $1.67 per pack (figures again quoted in US dollars by BAT),^49^ ^50^ and by 1995 the prices paid per pack of 20 of BAT’s top 4 brands (State Express 555, Embassy, Tusker and Diplomat) were $3.50, $2.90 $1.80 and $2.60 respectively.^51^ ^52^ Current (2008) retail prices of manufactured cigarettes on sale in Ghana range from the equivalent in local currency of approximately $0.85 to $3.50. Tax on tobacco products in Ghana is high in comparison with other consumer products, but low in relation to tobacco products in many developed countries, currently accounting for approximately 25% of the retail price of premium brand cigarettes such as State Express or Rothmans King size.^53^ According to the WHO, cigarette prices in Ghana are still low in relation to many other African countries.^54^

### Smuggling

Cigarettes have been smuggled into Ghana from its eastern neighbour, the Republic of Togo, for many years.^30^ ^55^ ^56^ References to smuggling are common in the documents we researched, as are quoted figures estimating the market share of smuggled brands, but these estimates are highly variable and their provenance unknown. However the market share of smuggled brands was probably highest in 1977, with estimates consistent at around 20% to 25%,^37^ ^57^ is reported to have fallen after the establishment of ITG, and to have remained relatively low until the present decade. By 2006 however the estimated market share of smuggled cigarettes was as high as 17.5%.^58^^–^60 Bond Street and Gold Seal, both of which are marketed in Togo but not Ghana, are now the two most popular smuggled brands.^53^ ^61^

### Advertising

Data on industry spending on advertising and promotion are few, but 1978 PTC was spending less than 1% of total income on advertising,^37^ reportedly because PTC had no commercial competitors in Ghana and the available supply of cigarettes was insufficient to meet any increased demand advertising would generate.^20^ ^38^ ^45^ In 1982 the Government imposed a directive banning all cigarette advertisements on state television, radio and in printed media,^62^ and all tobacco billboards were taken down. The industry response included attempts to counteract health concerns by publicising economic benefits of the industry,^63^^–^66 reduce the frequency of antismoking and anti-tobacco articles and place pro-industry articles in the media,^67^ ^68^ and increase networking to involve senior management in committees and associations providing contact with Government officials and business leaders.^49^ ^69^ BAT also used donations, and programmes of sponsorship for beauty pageants, sports and cultural events to generate good publicity in the press, television and radio.^19^ ^63^ ^68^ ^70^ For example, in 1990 BAT reported that:

“The company…sponsored the national dance championship competition under the Great Embassy Triple-Do…in all these the company received very good publicity in the press, TV and radio”.^69^

However, adherence to the advertising ban has been good, and the main forms of advertising that persist in Ghana are those on the vehicles of cigarette distributors, at street side vendors and other retail outlets, and on company paraphernalia and promotional items.

### Tobacco control policy and resources

The first Ghana Committee on Tobacco Control (GCTC) was established in 1993,^60^ and there is currently a national steering committee mandated by the Ministry of Health to oversee tobacco control activities, including media campaigns, health promotion and lobbying for smoke-free legislation.^68^ ^71^ However the resources provided for tobacco control activity are low, now amounting to funding for one person working in the non-communicable disease control unit. There is no specific budget for tobacco control activities.

Ghana was the 39th country to sign and ratify the FCTC, but implementation has been slow.^72^ A bill to prohibit smoking in public places was submitted to cabinet in 2005 but has made little progress since. There are no controls on cigarette sales.

### Tobacco consumption in other African countries

**Figure 3:**
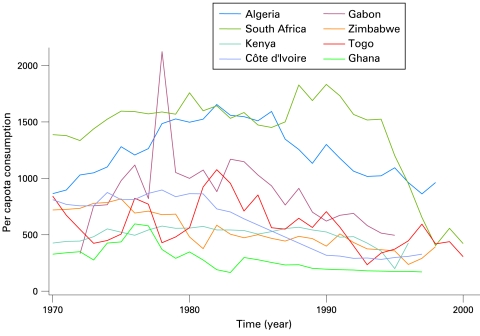
Annual per capita consumption of cigarettes in selected African countries with higher gross domestic product (GDP) in comparison with Ghana.

Tobacco consumption data available for African countries with higher gross domestic product (GDP) than Ghana^43^ are shown in fig 3. The data suggest that consumption has also fallen in these other countries over the past two decades. Of these countries, only the Republic of South Africa implemented an advertising ban (in 1999) in the period for which data are available.^73^

## DISCUSSION

The objective of this study was to document the evolution of the tobacco industry in Ghana, and to look for potential explanations for the low reported prevalence of current smoking. In the context of Africa, Ghana is a country at high risk of involvement in the tobacco epidemic, being populous, having enjoyed sustained strong economic growth, and having had a strong international tobacco industry presence in the country for more than 50 years. Despite this, after an initial surge originating soon after World War II and continuing until the mid 1970s, tobacco consumption has since fallen substantially. This and the reported low current prevalence of smoking^14^ ^74^ indicate that Ghana is an example of a country in which the typical tobacco epidemic appears, at least to date, to have aborted.

The information on industry activity in Ghana available for our study was limited predominantly to that available from the BAT archives. Since we have no external or independent sources with which to validate most of the information obtained the reliability of the data is unknown.^55^ ^75^^–^78 However, at times internal company document information was supported by contemporary news reports also held in the archive or by consistency of findings in more than one source. We are also limited in the reliability of our assessment of smoking prevalence in Ghana, since only one nationally representative study has been published.^14^ We have therefore had to rely on per capita consumption as a marker of likely trends in prevalence. However our research points towards three key factors that appear to have contributed to preventing a sustained increase in tobacco consumption after the mid to late 1970s. The first was that foreign exchange and domestic tobacco leaf shortages in the 1970s prevented the industry from responding fully to rising demand for cigarettes by increasing domestic production. That cigarettes were in short supply is evident from the fact that at this time, cigarettes were selling for up to four times their official retail price. The second was that at around this time the industry was subject to increasing government intervention, culminating in the government taking a 40% stake in PTC, and requiring a further 15% of the company to be sold to the public, in 1976. While we have no direct evidence that this change impacted on the efficiency and profitability of PTC, the relegation of BAT to minority ownership at such a key point in market development is likely, at least temporarily, to have inhibited investment and growth. The third was the implementation of an advertising ban in 1982.

Before 1976, PTC appears to have spent relatively little on advertising in Ghana, perhaps because of their monopoly position (BAT adopted a similar strategy in Kenya, where they enjoyed similar market status)^79^ but also because production was limited and could not meet the increased demand that advertising might stimulate. Advertising might have been expected to increase after the entry of Rothmans into the legal market through ITG in 1976, as a means for both companies to protect or expand market share; and again in the 1980s, when the tobacco leaf supply shortages that had restrained production during the 1970s and early 1980s ended and production could again increase. That leaf supply shortages had ended by 1986 is evident from the fact that Ghana then began to export tobacco leaf; at this point, presumably, leaf supply was no longer restricting cigarette production and in the presence of advertising to stimulate demand, domestic production could have increased to meet higher consumption. However at this stage, where growth was again possible, the advertising ban was in place and although not fully comprehensive, removed all billboard and media advertising. From this period, while advertising in Ghana continued at point of sale outlets, on distribution vehicles, and through sponsorship and other industry manoeuvres to obtain media coverage, the ban removed the major modes of product promotion at a time of particular commercial need.

It is therefore possible that production shortages account for the fall in consumption through the late 1970s and early 1980s, and that by the time the leaf shortages had eased in the 1980s, the advertising ban was in place and prevented the industry from stimulating fresh demand for the product it again had capacity to produce. Although the increase in consumption that appears immediately following the advertisement ban in Ghana may just be an artefact in the general trend of decline, it is also possible that the industry manoeuvres to counteract the effect of the advertisement ban could account for it. However, while this hypothesis accounts for the consumption pattern over time in Ghana, and the low overall level of consumption in Ghana relative to other relatively affluent African countries, it does not explain why consumption has also failed to grow substantially in recent years in several other countries without advertising bans, indicating that other factors are also important. Only one other African country for which data were available implemented an advertising ban during the period of comparison, and that was the Republic of South Africa in 1999. Although consumption in the RSA fell markedly in the late 1990s this occurred in the years immediately before the advertising ban was implemented, suggesting that pre-ban publicity arising from sustained anti-tobacco campaigns^80^ had an important effect. There had been no such campaigns in Ghana.

Official price changes appear unlikely to have played a major part in the trend in tobacco use in Ghana, not least because cigarettes have at times traded at prices substantially higher than the official price. Thus, although tax has been levied on cigarettes in Ghana for many years at a high rate in relation to that on other consumer products, the current price in US dollars paid (that is, the real rather than the nominal price) for cigarettes has changed little since the 1970s, and in real terms therefore has fallen substantially. Perhaps consistent with this, and in absence of reliable data, it appears that consumption of smuggled cigarettes has also not increased in Ghana since the mid 1970s. While the closure and relocation of BAT’s activity in Ghana may also be driven by other broader commercial reasons, a failure to grow substantially in this country might also be an important factor in that decision.

**What this paper adds**

- Constraints on industry growth arising from foreign exchange shortages and the taking of the industry into majority government/public ownership at a time of rapidly increasing demand in the mid 1970s.
- The imposition of a comprehensive advertising ban soon afterwards, in 1982.

Other tobacco control activities in Ghana are also unlikely to have played a major role, at least until relatively recently, in preventing the emergence of the smoking epidemic. Tobacco control activity during the 1970s and 1980s, when consumption was falling, was minimal. The first formal tobacco control committee was not established until 1993, and investment in tobacco control resource remains low. The ratification of the FCTC and promotion of the case for smoke-free legislation and other FCTC policy implementation represent considerable recent achievements however.

Overall our findings indicate that in Ghana, as in many countries,^81^^–^83 the tobacco industry typically survives hostile economic and political climates, and works hard to create a good corporate image and avoid measures that might impact adversely on demand for their product. However these efforts have not succeeded in achieving high levels of consumption in Ghana, either in absolute terms or in relation to other affluent African countries, where strong growth in consumption also occurred in the 1960s and 1970s^73^ ^84^ and continued after the peak in Ghana, before falling in more recent years.^73^ In Ghana in particular, a combination of factors including economic conditions that restricted industry growth and an early ban on advertising appear to date to have averted the major smoking epidemic and consequent toll of death and disability that would otherwise have been expected to unfold. While the relative contributions of these two influences are hard to establish, the overall low consumption levels at all times in Ghana relative to other richer African countries, before and after the advertising ban was introduced, suggests that early restrictions on growth of supply may have been particularly important in determining subsequent consumption levels.

Public health practitioners and health policy makers should be aware of the ever-increasing strategy of the industry to perpetuate their activities, and continue to engage with government to advocate for strong legislation to deal effectively with the industry. Ghana’s experience suggests that epidemic spread of smoking is not inevitable, and that controls on promotion, availability and advertising are likely to be effective in prevention in other countries. If effective legislation backs the current position, Ghana would be well placed to prevent an escalation of the current situation, becoming a model in that process.
